# Relational Digital Agency: An Everyday Life Study of Mobile Communication in Nursing Homes

**DOI:** 10.1177/20501579251379751

**Published:** 2025-10-08

**Authors:** Sarah Wagner

**Affiliations:** 7709 Department of Sociology Canada, University of Victoria Victoria, Canada University of the Highlands and Islands Inverness, UK

**Keywords:** Digital agency, older adults’ mobile communication practices, digital ageism, relational agency, social context of nursing homes

## Abstract

The pervasive association of long-term care with frailty and dependency has shaped research agendas. Everyday life studies that take into account care home residents’ knowledge, values, and experiences are few and far between. This research engages care home residents in dialogue to co-produce understanding about their lived experiences with mobile technologies. Drawing on qualitative research with 39 care home residents at long-term care sites in Canada, the paper calls for reframing digital inequalities in terms of relational digital agency. The analysis describes how meaningful communication environments in long-term care involve a wide range of factors, including effective access to analogue media and wider support networks, which enable residents to put meaningful limits on their uses of mobile devices. Moreover, the findings show how having the opportunity to deny and contest mobile technologies can be an important part of feeling socially and digitally included, which brings question to existing measures of digital inclusion that focus on quantity and quality of technology use. Whereas most research on digital agency has concerned youth, this paper develops an understanding of relational digital agency to account for long-term care residents’ experiences negotiating and adapting to digital change.

Agency is a contentious concept with relation to nursing homes, both from an academic and a policy/practice perspective. The so-called “fourth age” has become so strongly associated with frailty and dependency (Gilleard & Higgs, 2017) that research agendas on ageing care contexts risk contributing to ageist narratives whether they employ *or* contest notions of agency. This reflects an ongoing dilemma within critical gerontology relating to how and whether the concept of “agency” can be ethically and meaningfully engaged with. While there has been interest in reclaiming speak of agency in ageing research as part of wider agendas to revalue older adults’ subjective experience (Wanka & Gallistl, 2021), there also has been longstanding hesitancy in critical gerontology to ascribe significance to “agency” for risk of contributing to narratives that exclude and devalue subjectivities that do not conform with mainstream conceptions of agency (Katz & Calasanti, 2015; Van Dyk et al., 2013). Furthermore, focusing on individual action and agency may shift attention away from the institutional contexts that produce vulnerabilities in later life, including heightened experiences of digital exclusion (Hebblethwaite et al., 2022).

While there is growing recognition that nursing home residents face digital inequalities (e.g., CSA Group, 2022), there is minimal research—whether quantitative or qualitative—that contributes to an evidence base on nursing home residents’ everyday uses of digital technologies (Schlomann et al., 2020; Seifert & Cotten, 2020). This research gap may in part reflect ageist assumptions about nursing home residents’ incapability to contribute to research—most studies have been *about* rather than *with* nursing home residents (Fernández-Ardèvol et al., 2017) and have employed models of technology adoption that fail to consider subjective experience (Neves et al., 2019). More generally, everyday life studies that consider older adults’ values for and experiences with technologies have been overshadowed by research questions framed around how technologies can improve ageing outcomes (Givskov & Deuze, 2018; Peine & Neven, 2019).

This research addresses what has been largely overlooked in mobile communication research: nursing home residents’ everyday, lived experiences with mobile communication devices, including tablets, smartphones, and smartspeakers. More specifically, the paper explores: How do care home residents experience agency with respect to mobile communication and what implications does this have for how we frame digital inequalities?

Drawing on qualitative research with 39 care home residents in Canada, the analysis considers the material and social factors that enable residents to experience a sense of control and ownership over their mobile communication practices. The findings show how meaningful communication environments in long-term care will provide opportunity for residents to deny and contest new technologies, bringing question to the inherent techno-optimism of digital divide discourses (see also Wagner, 2020, pp. 161–162). Whereas much research and policy attention on the digital inclusion of older adults has been concerned with the quantity and quality of technology use, this paper calls for a shift of attention to digital agency and the relational factors that support people to actively manage if, when, what, and how they engage with communication media. The paper develops an understanding of relational digital agency that provides conceptual space for addressing the intertwined nature of institutional and individual factors that shape experiences of digital inclusion/exclusion.

## Mobile Communication and Residential Care

Paradigm shifts in social gerontology have incited interest in the cultural aspects of older adults “doings” with mobile media (Givskov & Deuze, 2018; Wanka & Gallistl, 2018). Research on older adults’ online communities (e.g., Nguyen et al., 2022), digital leisure practices (e.g., Chiribuca & Teodorescu, 2020), and mobile activism (e.g., Blanche-T & Fernández-Ardèvol, 2022) have revealed variegated everyday interactions with mobile devices, thereby diversifying and defying homogenized and stereotyped portrayals of older adults’ digital practices (Fernández-Ardèvol, 2020). However, a “deficit model” of technology use (Wanka & Gallistl, 2021) remains influential in mobile communication research in residential care settings. The host of Information and Communication Technology (ICT) research in nursing homes has been framed upon on an “interventionist logic” (Peine & Neven, 2019) with interest in how new devices and services can impact old age health and wellbeing (Neves et al., 2019; Wagner, 2022).

Positive outcomes associated with using tablets and smartphones in long-term care include reductions in depressive symptoms (Lin et al., 2020) and increases in social interaction and intergenerational communication (Akhtar, 2024; Jones et al., 2013). Likewise, there has been increasing policy attention to ICT use in care homes, particularly following the pandemic, and in Canada, numerous recommendations and initiatives have aimed to improve and promote ICT access and use in care homes (Wilson et al., 2022), alongside growing concern that care home residents have limited or inadequate access to the Internet (CSA Group, 2022, p. 28).

While research on everyday ICT use among nursing home residents is limited, studies have shown that Internet penetration rates in care homes are low (Seifert & Cotten, 2020), and a large-scale quantitative study in Germany with 80+-year-olds found that the living situation was the most important factor to explain probability of ICT use, where older people living in residential care were less likely to use ICT than their community-dwelling counterparts with comparable individual characteristics (Schlomann et al., 2020). This points to the need for further understanding of the contextual factors that contribute to care home residents’ disengagement with Internet communication. Research with ICT “non-users” has provided insights into the variegated factors that contribute to situational limitations on technology use and into the ways older people negotiate digital change (Fernández-Ardèvol et al., 2017; Gallistl et al., 2021).

## Digital Inequalities, Ageing, and Agency

The term “digital divide” gained academic attention in the early 1990s to describe disparities in ICT access and use across countries, regions, and social strata (Hilbert, 2010). Insofar as the problem was initially conceived of as an access divide—the so-called “first level” divide (Scheerder et al., 2017)—the solution was increased technology diffusion. The term “digital inequalities” arose early on within digital divide debates to evade the inadequacy of a “divide” metaphor and account for inequalities of use among those with access (DiMaggio & Hargittai, 2001; Lythreatis et al., 2022). This marked the “second level” divide, which has drawn attention to a wide range of factors that influence quality and quantity of ICT use, with particular attention to digital skills (e.g., Deursen & Van Dijk, 2010). A “third-level” digital divide that concerns differential outcomes from digital technology use has been widely recognized (Scheerder et al., 2017; Van Deursen & Helsper, 2015), and discussions have arisen about a fourth-level divide relating to the wider context that enables technology use (Van De Werfhorst et al., 2022). This latter work has been explored with respect to childhood education studies, but parallel issues have been described in old age care settings where further attention to organizational support is needed for effective technology use (e.g., Wilson et al., 2022).

Age-related inequalities in respect to the first and second level digital divide have been well documented (Francis et al., 2019), and studies have drawn attention to the important role of family members in acquiring digital devices for older relatives (Selwyn, 2003) and providing support with device management and setup as “warm experts” (Martinez & Olsson, 2022). Along these lines, older adults’ digital repertoires have been characterized as interdependent with the digital skills of family members (Hänninen et al., 2023), which resonates with the concept of a “social envelope” outlined in childhood digital inequality studies (Talaee & Noroozi, 2019). While research on older adults’ and children's digital practices has highlighted the role of relational supports, the concept of “digital agency” has been most often used in research on youth and higher education contexts, with minimal attention to the social support networks that enable experiences of agency (see Siddiq et al., 2024).

Drawing largely on research with youth, Passey et al. (2018) have presented an initial definition of digital agency as “an individual's ability to control and adapt to a digital world” (p. 426). This resonates with longstanding approaches to digital inclusion that consider how individuals can obtain “a degree of control and choice over technology and content” (Selwyn, 2004, p. 349). However, whereas Passey and colleagues’ conceptualization concerns the individual, framed around confidence, competence, and accountability for one's own actions, Selwyn's analysis of meaningful use broadens the approach to the contexts that shape inclusion, including support networks.

The shared dimension of digital agency has been further developed to account for the experiences of rehabilitation professionals and their clients, where “agentic spaces” enable clients to accomplish digital tasks with the support of professionals (Silvennoinen & Rantanen, 2023). This characterization of digital agency builds on longstanding discussions around “agency” as fundamentally relational and as a product of social interactions (Burkitt, 2016).

## Research Design and Methods

Qualitative research was conducted at four long-term care sites on Vancouver Island, B.C., Canada, involving 39 residents, seven staff, and five family members. The methodology combined reflective and creative sessions—to co-produce understanding about lived experiences—with mapping activities to identify pathways to change. Methods included in-depth interviews, digital storytelling workshops, collaborative reflection meetings, and asset mapping workshops. Due to ongoing concerns with COVID-19 infection prevention during the fieldwork (03/2022–01/2023), all research activities were conducted remotely, by videocall or phone call. The study was limited to include residents who experience no or mild cognitive impairments following a scoping study showing that within policy and research about nursing homes this group is often overlooked (Wagner, 2022). Ethics approval was granted by the University of Victoria and Vancouver Island Health Authority, and all research participants provided informed, voluntary consent.

This paper focuses on findings from the in-depth interviews (with 34 residents) and asset mapping workshops (with nine residents) with relation to both using and not using mobile technologies. The interview procedure was conducted in two parts and combined narrative inquiry (Jovchelovitch & Bauer, 2007) on social and communication practices through the life course with the mapping of communication networks and resources, or “communicative ecologies” (Hearn et al., 2009). Interviews were on average 42 minutes in length and used visual aids shared on screen, including picture cards and mapping sheets developed in a preliminary study (Wagner, 2022). Participants ranged from 50 to 103 years of age, with an average age of 77 years. The asset mapping workshops involved a virtual tour of the care home's floor plan and invited participants to reflect on how different rooms and spaces enable/constrain social interaction and/or media engagement.

The analysis inductively moved from codes using the participants’ own phrasing to thematic categories and theoretical concepts. Following the initial emergence of a code hierarchy, the dataset from each participant was re-analyzed to explore contributing factors within individual participants’ standpoints. This profiled re-reading of data enabled connections to be drawn with wider contextual factors that shaped residents’ varied experiences with mobile communication. The analysis developed in this paper largely draws on the second phase of the analysis, with attention to participants’ narratives to develop depth of understanding on experiences of digital agency.

## Findings and Analysis

While all four care homes had tablets for residents to use and offered support with scheduled videocall appointments, nine participants had never videocalled before the interview, including four participants who owned their own tablet or smartphone. Residents’ reasons for engaging *and* not engaging with Internet communication were not simply about affordability or capability but reflected wider relations of support as well as identities and preferences.

**Figure 1.**
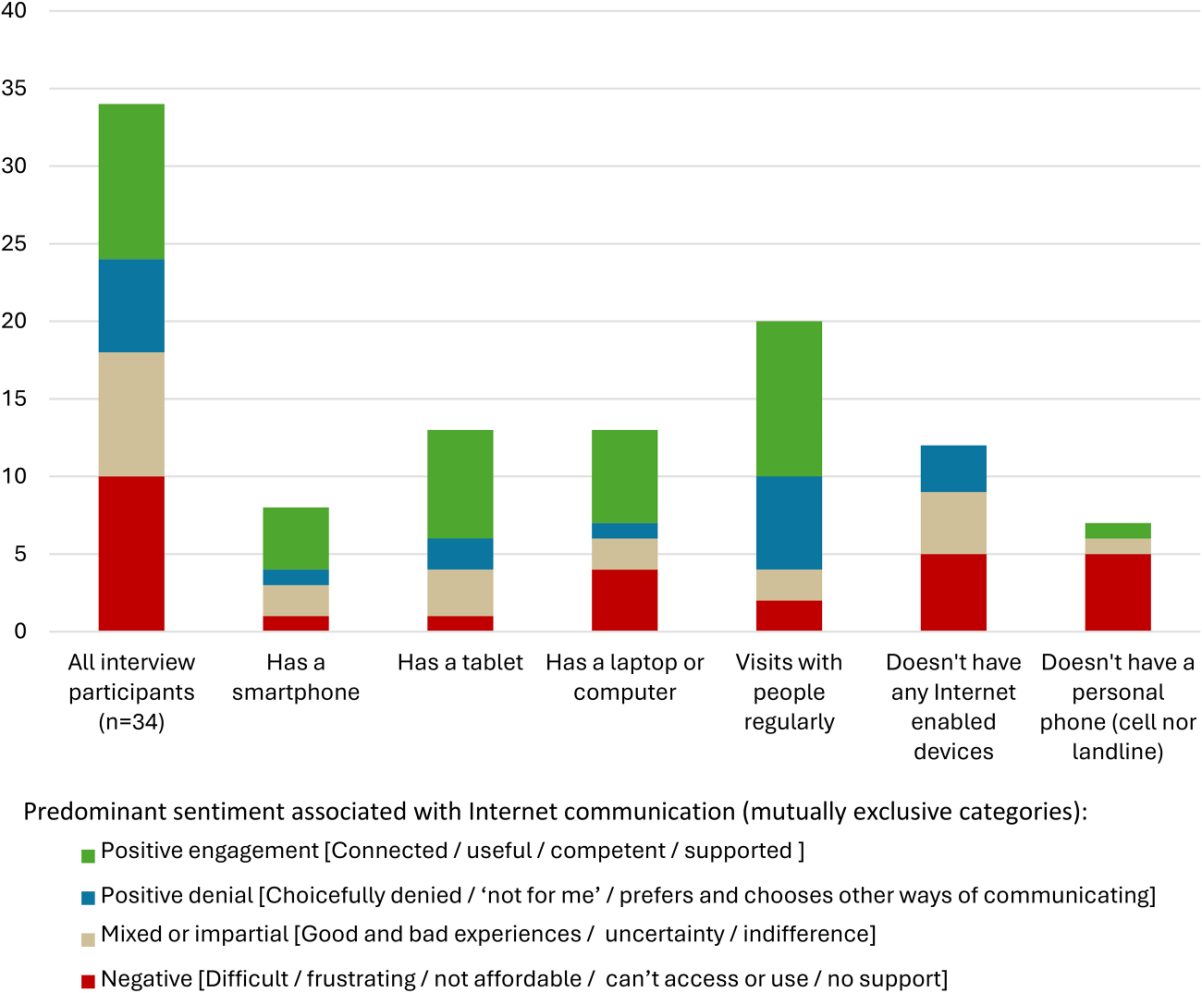
Key Aspects of Interview Participants’ Communication Resources in Relation to the Predominant Sentiment They Associated With Internet Communication.

Figure 1 summarizes interview participants’ differential access to communication devices and resources in relation to how they generally felt about Internet communication. Participants expressed a range of emotions when speaking about digital technologies in respect to the immediate context, their longstanding communication habits, and the available supports. As can be seen in Figure 1, all participants who spoke predominantly positively about (either adopting *or* denying) Internet communication had regular contact with people outside the care home. In what follows, the analysis is developed in line with the classic levels of the digital divide (Scheerder et al., 2017)—(1) access, (2) use, and (3) outcomes—and in this way explores the mediating role of relational aspects (such as outside support) across all three levels.

### Accessing Mobile Devices—From Relational Constraints to Positive Denial

Long-term care services in the province of B.C. typically leave residents with 20% of their after-tax income to cover all unfunded expenses, including costs associated with communication devices and services, personal assistive devices, personal transportation, and personal hobby supplies (Government of B.C., 2025). As shown in Figure 1, 12 participants did not have an Internet device; however, this was not simply because of the financial cost. While nine participants described not being able to afford the communication devices/services they wanted, these participants and others also described wider forms of exclusion relating to lack of access to finances and shops and lack of guidance on what to buy. Furthermore, six participants, including three who had a device, described how they had turned down an Internet device as they preferred other ways of communicating.

#### Without Outside support, it's Difficult to Get Tech

Residents who were not able to venture outside the care home or who did not have regular contact with visitors often did not know how to go about obtaining new communication technologies. For some residents, this was because they could not access personal funds. Seventy-seven-year-old Jared^1^ explained:

Theoretically [there would be spending money], but up until very recently, I didn’t have any money … I would have to go to the social worker, ask her to contact the public trustee and issue me some money. I haven’t had a dollar in my pocket for a year, nothing. So, I don’t have money. … I’d like to [have a tablet] but I don’t. I can’t afford one. (Jared, 77 years)

Other residents who could access money explained that the monthly spending allowance wasn’t sufficient to save up for a device. Fifty-two-year-old Jude explained:

I had one [a tablet] … But I pawned it off because I needed the money … I wasn’t able to get it back though, my tablet, that was the one thing I needed … In here, cigarettes, and we have to pay for cable. It's hard to get by in a month with that money. (Jude, 52 years)

Jude was one of 11 participants who had previously used the Internet but no longer had a working Internet device. Sixty-four-year-old Wendy explained:

I’ve had a computer, and I think it's in possession here [at the care home]. It was an Apple. I have an iPad, which I lent to a friend who dropped it in the water, and so it doesn’t work.

Wendy didn’t have any family members to help her source a device and she wasn’t sure how to go about asking for support and didn’t want to “bother” the staff.

Similarly, 71-year-old Donald had used the Internet regularly before moving into the nursing home, but now didn’t have an Internet-enabled device and didn’t know how to obtain one:

I would welcome any help I get. I just don’t know who to ask. I think that's the bottom line. I think they all have specific jobs. … And I don’t like to bother people needlessly, so I’m in this space now where I tell myself now every day, “Patience, you need patience,” and I let things work themselves out. (Donald, 71 years)

Having someone outside the care home to help source and set up devices was an important enabling factor, as participants explained that staff were “overwhelmed” with other tasks. While Donald was not in regular contact with family, other residents, like 84-year-old Thomas and 77-year-old Harold, had family who sourced their devices, brought in materials, and reconfigured things when needed.

#### Access Isn’t enough—Personal devices are Important for Privacy and for Daily Habits

Participants described challenges when accessing and using the care homes’ devices. The new digital practices adopted by the care homes during the pandemic did not support personalized access to the Internet as the focus was on scheduled videocall appointments. Sixty-nine-year-old Ronald relied on assisted videocalls using the care home's tablet to contact his daughter abroad. He had no family in Canada and, like Jared, could not access his finances to purchase a device. Using the public phone was difficult as there was no privacy, and he wanted regular access to the Internet so that he could continue to learn about languages and history.

Outside of assisted videocalling sessions, limited Internet access was provided at the largest site via one Internet-connected computer shared among 300 residents. Before moving into the care home, 50-year-old James had used the public library's computer to contact family abroad, but the library was now too far away. He had an “old … little cell phone” that could get on the Internet but was “so small it's hard to see.” After covering the cost of a powered wheelchair—which enables him to navigate uneven pavement and head into town—he doesn’t have enough money for a tablet: “The money in here is pretty tight … I just wish that we had like tablets for example so that we wouldn’t have to sit in line for one computer.” Having a personal device was not only important for privacy but also for providing flexibility with respect to when and where devices were used, which supported some residents to establish valued routines for reading, watching, and talking on the phone that aligned with their longstanding communication and information-seeking habits, as also found in preliminary research conducted in person (Wagner, 2022).

#### Not Wanted—Smartphones are Tedious or Invasive

Some participants described their dislike of specific affordances of smartphones, such as being constantly available and feeling the need to respond or being suddenly on camera. Eighty-four-year-old Kenneth has a tablet that he uses for watching YouTube and looking up information about boats, but “stays away” from the smartphone his daughter gave him because he doesn’t want to get involved in messaging: “It gives me an obligation to reply … It is easier to use the landline as far as I’m concerned.” Similarly, 88-year-old Susan evades the pressure to use a smartphone as she feels she has enough technology in her life:

A lot of my friends have iPhones, and I said, “Oh Lordy, No!” I’m not going to get into iPhones. I just think, “Oh Lordy,” I have my own Internet and my own landline phone, and my own printer, and scanner, and my own computer, and my own iPad there—Lordy, I mean, and I’ve learnt all this since 2011. (Susan, 88 years)

Susan has a long-distance calling plan on her landline phone, which she uses to connect with an old friend for “interesting discussions … especially about politics.” She drew the line at getting an iPhone as she felt fatigued with new tech and wasn’t interested in instant messaging.

Like Susan, 88-year-old Shirley and 73-year-old Jules associated messaging devices with tedium. Shirley explained that tablets were not for her as she didn’t want to be “looking for any messages on it,” and 55-year-old Jordan positively described how he managed his life without needing to be always contactable:

I never wanted a cell phone … it was too hard to handle with me being in a wheelchair … so I choose to have a landline and I do my phone calls before I go out and I’m quite organized … I arrange things and set them up vocally rather than on the keys on the keyboard. (Jordan, 55 years)

Jordan was “out and about” a lot, using the public bus service to head into town, and considered the nursing home as “just a place to sleep and a mailing address.” Having access to public transport and being able to see people outside the care home meant that online messaging was not needed or even desirable.

#### Not wanted—New Tech Might Interfere with Preferred Ways of Communicating

Emotional attachment to in-person, more traditional, or “simpler” forms of communication was a recurring theme among participants’ motivations for not wanting to have Internet devices. Ninety-six-year-old Annette emphasized that she enjoyed reaching out to people in person and that this was what made her feel connected. She explained, “Generally, I’m just always talking to someone.” Similarly, 97-year-old Lucy was not interested in using the Internet or videocalling, as she knew “nothing about that” and emphasized that she was “more interested in helping others” and caring for them in person. For communicating by distance, she preferred her landline phone, as she could use it independently to talk with her niece. Eighty-four-year-old Agnes also prefers voice calls on her landline; she wasn’t interested in videocalling as it seemed a “little phony,” as if the other person on the call wasn’t “actually looking at [her].”

Sixty-two-year-old Philip has a basic cell phone that he uses for voice calls, and occasional texting: “I bought it at [the pharmacy]; that's it, easy and simple.” When his daughter offered to send over a computer or tablet for Christmas, he turned it down. He explained,

I told her, “No, don’t bother,” because the thing is as I see it, if I get interested in a computer or laptop or tablet or anything, I’ll probably spend more of my time on that than what I’m doing now. And I like what I’m doing. I like going, visiting, and stuff like that. … It's totally better [seeing people in person]. (Philip, 62 years)

Philip could easily access bus routes—“I take the bus all the time … I go all over”—and would visit family members’ houses regularly, often cooking meals for them. He didn’t want a tablet as it might mean he would see people less in person. Participants like Jordan, Susan, and Philip had family support networks and/or the means to head into town independently, which enabled them to be selective about how they wanted to communicate.

### Everyday Use—From Frustrations to Spheres of Competence

ICT use in nursing homes, and among older adults more generally, has been framed around discourses about incapabilities and individual barriers to technology use (Gallistl et al., 2021). While participants sometimes reproduced negative stereotypes about ageing and digital technologies, their narratives also revealed how having access to a range of devices and outside support provided space for positivity and a sense of choice over how they engaged with Internet media.

#### “In Limbo”—Digital Tech is Frustrating When There's No Outside Help

Residents described frustrations with mobile devices when they felt they lacked control over platforms or devices and the type and frequency of support they could access. Sixty-two-year-old Barbara had decided to get a laptop on her cousin's advice but had only managed to use it for a couple days. She had enjoyed looking up “interesting stuff to read about meditation and stuff like that” but then it stopped “working properly.” She explained,

I just started getting it [the laptop] going. I wish someone could come in and show me what to do with that computer because it's in limbo again, and I just don’t know how I’m supposed to fix it really. (Barbara, 62 years)

Similarly, 65-year-old Hank expressed frustration with not being able to use his mobile devices because there was no technical support or training at the care home. He had never used a computer or the Internet before—“my wife used it all the time, I never used it”—but when he moved into long-term care, he bought himself a laptop and a tablet with the hopes of being able to look up information about cars, but the devices were “no good” to him:

I got a laptop, and one of these tablets but the thing is, they are no good to me because I don’t know how to use it. That's what I wish they’d have in this place, and I’ve mentioned it to a few people that they should have a course on computers … but like I say I don’t know how to use this stuff at all. (Hank, 65 years)

Similarly, 86-year-old Alexander was becoming exasperated with having to navigate different systems for videocalling, which was influencing his confidence levels:

I have a laptop but it's a different system [than this tablet] … We tune in every day to a different system, and I don’t understand it yet. I have them [the staff] tune it every time for me. I have no idea what to do … I’m stupid when it comes to setting it up. (Alexander, 86 years)

Alexander did not have anyone outside the care home to help him set up his devices; having to rely on overly busy staff made him feel like a burden, increasing his frustration.

Residents who had outside help spoke more positively about their experiences learning to use new technologies, even when they reproduced negative stereotypes about old age and digital capabilities. For example, 95-year-old Edna reproduced digital immigrant narratives (see Bayne & Ross, 2011) while at the same time speaking light-heartedly about learning to use a smartphone. She explained,

At our age, we didn’t really grow up with it … My granddaughter is a lot smarter on a computer than I am … I could never use the smartphone. I use that to call or if they’re calling, I’ll answer it. I can’t look anything up on this. I can do it on a computer, but that takes time … I had a bigger iPhone [iPad] and I couldn’t figure it out. I’ve gotten a little bit dumb [laughs], so I got one of these [smartphone]. And I don’t know how to pick up calls. I’ve got 28 missed calls on it … I’m technically challenged now, and I never used to be. I was so—don’t get old [laughs]. (Edna, 95 years)

Edna didn’t like asking staff to help with her smartphone—“You hate to ask them [the staff] when they’re all so busy”—but she had a workaround: she would call on professional help. She explained, “I usually get hold of [electronics store] who send out their computer man.” Being able to afford outside help enabled Edna to regain a sense of control over her smartphone, even though she characterized her technology use in terms of incapability.

#### Setting Limits on Digital Skills Learning is a Positive Experience

Positive sentiments were often expressed alongside descriptions of agency in decision-making over whether and what to learn about new devices. Ninety-six-year-old Annette associated her old age with the difficulties she experienced when learning to use new technologies, but generally felt positive about how she used her iPad as she had set limits on what she wanted to learn, which wasn’t anything “fancy-dancy”:

I’ve had a couple of, I guess, setbacks [using the iPad] but I can mostly just directly go online for just a little talk [videocall], but I don’t do anything fancy-dancy, you know, involved, on the iPad … They had a little club [to learn how to use the iPad], but I didn’t bother. I mean all I need is what I’m using it for. I don’t want to do anymore. Not at my age, not right now. (Annette, 96 years)

Other residents also conveyed positive sentiments when speaking about their decision to turn down opportunities to learn about new applications or devices, or more generally about their lack of knowledge of digital technologies and the Internet. Jade (aged 103) had never videocalled before as she didn’t feel it was relevant for her. She explained, “I find some of the technology these days is a little beyond me and perhaps it's my fault that I can’t see it applying to me.” She had recently acquired an iPad from her daughter who set it up for reading books. She wasn’t interested in learning to use the iPad for anything else as she had her TV for news—“I’m a newshound!”—and her landline for making calls—“It's worked out very well. I can make phone calls to [Europe] by myself and manage to talk to the rest of the family over there.” The iPad was the device she would most often need help with, but as her daughter dropped in daily, any issues were sorted promptly: “If I get in a muddle [on the iPad], she’ll get me out.”

Like Jade, Lucy was confident in her decision to set limits on what was worthwhile knowing or learning about Internet media. She spoke about how she wanted to spend her time making in-person connections to care for people. She had interest in continuing to improve herself in these ways and felt she didn’t need new technologies. She explained,

I never even thought about it [videocalling]. In fact, there's a lot of words I don’t understand. Somebody had some talk about the video [calling] here and I just can’t understand computers. So, I had to leave. I’ll just concentrate on what I need to do to change for the better. (Lucy, 97 years)

Philip also spoke with confidence, and a sense of pride, about his lack of knowledge about the Internet. He explained,

I don’t even know how to use it [the Internet]. That's weird in this day and age, isn’t it? … Most people are on it now 24/7 and I’m saying, “What are you talking about?” No, yeah, I don’t know nothing about it … My oldest daughter, she showed me how to text on the phone. That was only two years ago. It takes me a while to text, but I get there. I’m a one-finger text guy, you know. (Philip, 62 years)

As described above, Philip turned down a tablet as he preferred heading out to meet people. His minimal digital skills were not a deficit but something to be proud of as they were representative of his decision-making and “non-tech” identity.

#### Having More Options Creates More Opportunities to Feel Digitally Competent and Confident.

Participants like Anette and Philip, who expressed positivity about the limits they set on their digital skills, had access to a range of communicative resources—including regular in-person visits or access to transport. Feelings of digital capability were often discussed in relation to having access to multiple devices and ways of communicating to which different functions could be assigned. Six participants who explicitly emphasized capability when using a tablet and/or smartphone also had laptops, landlines, and/or regular in-person visits. While Kenneth “stays away” from the smartphone as described above, he doesn’t need any help using his tablet to search the Internet: “The tablet's fine for what I use it for.”

Similarly, 84-year-old Rupert was competent using his tablet: “I usually do it [join online church meetings by Zoom] on my own. And then I Skype [videocall] with my wife, and I connect on my own.” Rupert described how he navigated between an array of devices to stay connected: his laptop is for emailing, his landline for talking with his sister, and his tablet for videocalling, including Skype and Zoom depending on the context. Rupert struggles with manual dexterity and his tablet was difficult at first. “I have trouble using [the tablet]. It's impossible to write something. The wrong thing comes out. It's horrible. I type on the desktop.” His sister helped him source the tablet, and while it took him a while to get used to it, it became an important part of his media repertoire. He emphasized, “I need them all!” Unlike descriptions of polymedia contexts where users seamlessly move between a wide range of interrelating affordances on their smartphones (Madianou & Miller, 2013), participants often developed workarounds to digital barriers and limitations by designating singular functions for their devices. For some residents, like Rupert, this meant that having an array of devices was important to maintain communicative preferences while also adapting to changing abilities.

### Everyday Outcomes—Reconnecting, Leisuring, and Feeling Challenged

Many participants described established routines for connecting with family and friends, often involving in-person visits and landline telephones. While mobile devices played an important role for social connections for some participants, they were often valued for providing opportunities for self-exploration and growth: Residents described how they could reconnect with media, seek out information, and take on new challenges. These outcomes were possible when residents had access to outside social worlds and/or could source and effectively use preferred devices and services—this often involved having a family member who regularly visited the care home to bring in supplies and maintain devices.

#### Adapting to Changing Abilities and Taking on New Challenges

Residents who found it difficult to use a touchscreen device or see a screen described how having an assemblage of devices, including smartspeakers, TV screens, tablets, and laptops, helped them adapt to changing abilities. Harold, for example, used his smartphone only for photography and his laptop for other tasks as he could not type on the touchscreen. He explained how his smartphone, laptop, and a good relationship with a volunteer all formed a key part of his setup:

When you have [a disability] actually, you shake, and it's pretty difficult to get good quality pictures. So, I would use a smartphone and get [the volunteer who puts on a photography session] to go take a picture with the phone, and then bring it back in my room and put them all on the laptop, and then I like to edit them. (Harold, 77 years)

Harold's sister had an important role—she would come in weekly with more supplies when needed, including photo paper and ink cartridges. In addition to editing photos, Harold uses his laptop to look up information and message people—“I do all the technology from the desk here.” As it is becoming more difficult for him to hold books and see small, printed text, he is reading “more and more … all on the Internet.” For Harold, the technologies he uses are an important part of what makes the care home feel “gilded.” He explained,

A gilded cage is the way I felt at first because I wasn’t used to the institutional environment. And as I got more used to it, the cage aspect disappeared, but the gilded part is still there … The technology makes the environment much more challenging and interesting than it would be without it. It expands my world back to kind of what it used to be. (Harold, 77 years)

Similarly, Thomas explained that his daughter had “really helped a lot” by finding workarounds that enabled him to stay connected as his vision deteriorated, such as hooking up his iPad to his TV:

It got where I could punch in the stuff on the iPad and get a picture up on the big screen … and we could see each other, and she could show me around whatever she's been doing. It was really nice. (Thomas, 84 years)

Before the pandemic, Thomas thought of his television as his “best friend”; he had never used the Internet, nor was he interested in doing so. The combination of social distancing measures and the deterioration of his eyesight had made him increasingly turn to Internet communication. He explained, “I’ve got a lot of stuff here entertainment wise to keep me busy. And the more my sight goes the more I’ll use this thing [the smartspeaker].” He explained further,

Well, we got [the smartspeaker] because my sight was going, and we thought, “Well, if I can’t read this stuff, I better be able to talk to someone.” So, I can talk to this thing, and I can get all the information I want … And it's really, really good. Like yesterday I got a station on—I think it was a YouTube station coming through there. Really a good station, all the music I liked was playing. I was really happy about that. (Thomas, 84 years)

Grace also has a vision impairment. She had been an avid reader and word game enthusiast and now didn’t want to lose the ability to spell. Having a smartspeaker had allowed her to challenge herself with word games and spelling tests. Her family had set the smartspeaker up for her, but now it was broken, and the staff “couldn’t get it going.”

#### Reconnecting with media and social interests

With family support, Thomas and Grace had been able to reconnect with media that were meaningful for them, but ongoing support would be needed for Grace to continue to use her smartspeaker.

Like Thomas, James described nostalgic enjoyment when accessing media on a mobile device: “I am just using my telephone to pick up tunes that I remember from being a young pup that I used to listen to.” Similarly, Susan described how photography made her feel connected with her youth: “The first item I bought with my own money [in her youth] was a sewing machine and the second one was a camera.” At the care home she joins a photography course by videocall and has thousands of photos stored on her computer. She explained, “I get a lot of pleasure just flipping through—I have my computer on quite often, with my family pictures.” Her computer was also important for maintaining civic connections. She’d been an “advocate for others” throughout her life and now used email to keep “involved in social justice stuff and politics” and “sign petitions.”

Having more personalized access to media enabled some residents to reconnect with social worlds that were important for them, expanding their world back to “what it used to be,” as Harold put it. These engagements were often supported by access to transport, finances, and shops, and/or regular support from a family member or friend.

Residents who didn’t have regular visitors and who couldn’t leave the care home independently could benefit from Internet communication, yet often had no way to buy, maintain, and effectively use a device. Wendy, for example, was formerly an avid Internet user, perusing chat rooms, and looking up information about philosophy, spirituality, and current events, which was something she missed talking about: “People here aren’t able to discuss things the way I would want to discuss them—Russia, Ukraine, for example—and it's tough sometimes.” She struggled to find anyone at the care home who understood her or took her seriously, and she felt that she was “not being treated quite as bright as I am” by staff. Similarly, Wayne explained that he didn’t know how to approach staff and didn’t want to bother them but wanted to take on new challenges and relearn how to use a computer. Ongoing, close relationships with staff who could provide designated support with both sourcing and maintaining devices would be important for residents like Wayne and Wendy to benefit from communication media.

## Discussion—Reframing Digital Inclusion and Agency

Participants’ everyday experiences with mobile communication were diverse in respect to the devices and platforms they engaged with, the uses they designated to different tools, and their associated emotions, which spanned frustration, nostalgia, humor, isolation, and competence. These findings support other studies that draw attention to the diverse ways that older adults engage with mobile media (Givskov & Deuze, 2018), countering homogenizing portrayals of older adults as technologically incapable or averse, which have particularly influenced ageing and technology research agendas in long-term care (Wagner, 2022). As also found by Gallistl et al. (2021), a wide range of contextual situations shaped the ways that individuals engaged and didn’t engage with mobile devices. For example, preferred videocalling platforms depended on who one was speaking with, and devices that caused frustration in one situation became valued in another when outside help was available.

Whereas other studies show how physical disabilities present challenges for the effective use of digital devices (Snyder, 2023), participants in this research more often described how new technologies and arrays of different tools provided avenues for them to adapt to their changing abilities, such as smartspeakers that could be used for literary games when one's eyesight worsened or the assemblage of a smartphone and laptop that allowed for photo-editing when one's hands became shaky. Feelings of failure were not directly associated with technology disengagement (cf. Gallistl et al., 2021) but rather arose when other communicative options and/or supports were not available. Moreover, participants often associated positive sentiments with digital disengagement: residents described pride when refusing new technologies or when choosing what they didn’t want to learn about a device.

Situations where residents attached positive sentiments, including pride and humor, to their non-use of mobile devices were intertwined with access to outside support. Support networks included other tools and modes for communicating—bus routes, private landline connections, powered wheelchairs, and family visits—that enabled feelings of competence and confidence about everyday communication practices, and thus the space to say “no” to new devices. For example, Philip, who regularly used public transport to meet with family, felt positive about his decision *not* to use Internet communication as he had effective access to other communication resources.

Other studies have also found that older adults who identify as “offliners,” like Philip in this research, do not always perceive their non-use of the Internet negatively or as a form of exclusion (Seifert et al., 2018). This research contributes further understanding on how relational factors, including effective access to analogue media, enable positive experiences of ICT non-use, and moreover, that the capacity to deny ICT can be an important part of feeling socially and digitally included. That is, residents’ narratives in this research show that having outside support networks and effective access to other forms of media, including transport and face-to-face encounters, can provide the grounding upon which meaningful engagements and disengagements with digital technologies can transpire.

These findings point to two key implications for how we understand digital inequalities. First, digital inequalities are not simply about differing levels of technology access or use, but more fundamentally about differing levels of agency over communication and information practices. Accessing mobile devices can increase frustration when no support is available, whereas denying unwanted forms of mobile communication can be an important part of a meaningful communicative ecology. A more comprehensive understanding of digital inclusion is needed that can account for the important role of technology disengagement when communication devices and services are unwanted. While the third-level digital divide shifts attention to inequalities in the outcomes of communication technology use (Scheerder et al., 2017; Van Deursen & Helsper, 2015), there remains an underlying assumption that all people *should* use and obtain successful outcomes from digital technologies.

Second, the findings show how experiences of digital agency result from material and social interactions. When participants had a wider range of communication resources and options—including access to transport, landline phones, multiple devices, and regular in-person visits—they had more agency over how they engaged with mobile communication, choosing when, where, and what forms of communication they wanted to use or deny. This builds on existing work on “agentic spaces” (Silvennoinen & Rantanen, 2023) and “social envelopes” (Talaee & Noroozi, 2019) that describe how support networks enable meaningful engagements with digital technologies. This study contributes new understanding on how these supportive “spaces” involve wider contexts of media use, including analogue forms of communication, which can play a fundamental role in shaping emotional responses to digital technology use. Having “warm” digital support from family or friends and/or the ability to access outside social worlds enabled participants to have a wider range of communication resources.

Residents who did not have these supports described feelings of isolation and disconnection, and the prospect of relying on staff for support increased frustrations with communication devices, as described by Wayne when trying to be “patient” and Alexander who did not want to feel like a daily burden when setting up video calls. Overcoming such barriers at care homes would not simply be about digital skills development or further access to devices. Participants’ experiences suggest that digital support at care homes needs to be more holistic and better grounded on personal relationships, with further attention to how staff can provide “warm” support with identifying and setting up relevant communication resources—including analogue media and in-person engagements, sourcing new devices, and providing ongoing, caring support with maintaining and using devices.

### Limitations and Delimitations

The research was initially designed to be conducted face-to-face, including participant observation. Remote research presented challenges both for developing rapport with participants and for developing in-depth understanding of the everyday spaces and practices in the care homes. To mitigate this, the digital storytelling workshops and reflective meetings helped to provide further presence within the care home and most participants engaged in casual, friendly, and in-depth conversations with little attention to the virtual dimension of the interaction. However, some residents were less comfortable with audio/video calls, and some may have chosen not to participate for this reason, which has implications for the range of experiences this research reflects. Furthermore, it is important to note that this research presents an unbalanced understanding of the situation in long-term care as it was designed to address the situation for residents who are living with no or mild cognitive impairment and who form a minority within the long-term care system. Further research could explore experiences of digital agency among care home residents living with dementia, building on work on embodied and emotional forms of agency (Boyle, 2014).

## Conclusion

Whereas prior work on digital agency has largely concerned youth (Siddiq et al., 2024), this paper has developed an understanding of relational digital agency to account for long-term care residents’ experiences negotiating and adapting to digital change. The narrative and mapping approaches employed in this research respond to a gap in understanding on mobile communication in long-term care, as much prior research has overlooked residents’ subjective experiences. The everyday life focus of this research developed insights into residents’ experiences of digital agency, which have implications for digital practices in care homes and for digital inclusion research more broadly.

Findings showed how a greater array of analogue communicative resources and supports contribute to a more positive relationship with Internet media. More holistic and caring approaches to digital support—ones that encompass all stages of identifying and using a wide range of communication resources, including analogue forms of communication—will be important to address digital inequalities in care homes.

The research also contributes to digital divide debates by showing how meaningful communication environments afford space for people to deny and contest digital technologies. This finding brings into question existing measures of digital inclusion that focus on the quantity and quality of technology use and calls for further research attention to how people across institutional settings are afforded the capacity to deny unwanted forms of digital communication and how this capacity may underpin or contribute to experiences of digital inclusion.

## Notes

1. All participant names have been changed to maintain confidentiality.
